# Busulfan damages spermatogenic function by inducing orchitis

**DOI:** 10.1371/journal.pone.0322721

**Published:** 2025-07-22

**Authors:** Lingjun Zhao, Kaihui Wu, Shiyuan Xu, Songqi Liu, Kaimin Yuan, Dong Wang

**Affiliations:** 1 State Key Laboratory of Animal Biotech Breeding, Institute of Animal Science, Chinese Academy of Agricultural Sciences (CAAS), Beijing, China; 2 College of Animal Science and Technology, Jilin Agricultural University, Changchun, China; University of Hyderabad, INDIA

## Abstract

In order to clarify injure mechanism of busulfan to spermatogenic function, we treated mice with busulfan, the testicular and epididymal weights and sperm concentration significantly decreased and the sperm malformation rate increased over time. Moreover, testicular interstitial cell infiltration, a smaller seminiferous tubule, and disorganized and shed spermatogenic cells were also observed by immunohistochemical, immunofluorescence detection after the busulfan treatment. Furthermore, the enzyme-linked absorbance assays showed serum interleukin (IL)-6, IL-1β, and tumor necrosis factor-apha levels (inflammatory factors) were significantly upregulated; blood-testis barrier (BTB)-related protein levels (e.g., N-Cadherin, occludin, and connexin 43) and vimentine gradually decreased. So we infer busulfan treatment induced orchitis, further disrupted the BTB and disrupted the spermatogenic microenvironment, then decreased vimentine and gradually damaged the cytoskeleton, which cause spermatogenic cells losing their supporting from sertoli cells, androgen regulation was also affected, which was detrimental to spermatogenesis. The study result will improve the efficiency and safety in spermatogonial stem cell transplant recipients.

## Introduction

Busulfan (1,4-butanediol dimethyl sulfonate) is a leukemia treatment that efficiently destroys endogenous germ cells and thus has been widely used to prepare recipients for spermatogonial stem cell (SSC) transplantation [1,2]. However, it affects hematopoietic function, resulting in growth retardation in children receiving long-term treatment [3,4]. Furthermore`, busulfan damages the spermatogenic epithelium`, reducing sperm counts and sometimes causing sterility [5].Therefore, due to its high toxicity, busulfan severly affects male reproductive function, resulting in more risk for those receiving cancer treatment or preparing for SSC transplantation [6]. Thus, in-depth mechanistic studies could provide data to minimize busulfan toxicity and promote widespread use.

The blood-testis barrier (BTB) is a barrier structure composed of ectoplasmic specializations, tight junctions, gap junctions, and bridging junctions, promoting spermatogenesis and the maintenance of normal reproductive function by providing an immune-privileged environment [7]. These structures are also forms of intercellular adhesion junctions [8], which are crucial for supporting and regulating the interdependence and orderly arrangement of spermatogonia and Sertoli cells in the seminiferous tubule [9]. The cytoskeleton is primarily composed of actin filaments, consisting of microfilaments and microtubules, which are the main determinants of cell shape. During spermatogenesis, microtubulin and microfilament proteins collaborate to regulate the BTB structure, ensure spermatogenesis, and facilitate smooth development and transport to the lumen [10,11]. Once these structures are damaged, the environment for spermatogenesis is severely affected.

A previous study reported that testicular busulfan injections induced considerable cellular infiltration, similar to that in the interstitium [12]. In addition, if testicular immunity is disrupted due to trauma or infection, autoimmune orchitis (characterized by interstitial cellular infiltration) is possible [13]. Therefore, some researchers hypothesized that busulfan might impair spermatogenesis by inducing orchitis; however, studies have been unable to confirm this. According to our team’s previous research findings, the molecular weight of busulfan is very small and can freely enter the seminiferous tubules. It was detected in epididymis that after 30 min, busulfan through the BTB structure, and we found that testicular busulfan injections increased the levels of inflammatory factors [14]. Thus, we hypothesized that busulfan induced orchitis, but we could not identify how it impairs spermatogenesis. Others found that fluorine can also cause orchitis [15] and pro-inflammatory cytokine production, such as tumor necrosis factor-alpha (TNF-α), transforming growth factor-beta 3 (i.e., TGF-β3), and interleukin (IL)-1a, by Sertoli cell and germ cells, impairing spermatogenic cell development and differentiation [16]. Pro-inflammatory cytokines also disrupt connexin endocytosis, recycling, and degradation [17,18]and damage the cytoskeleton [19,20], suggesting that vimentin, found in the cytoskeleton of Sertoli cells, is essential for maintaining normal cell morphology and to the physical support and signaling regulation between and by Sertoli cells in spermatogonia [21,22]. Androgens expressed by mesenchymal cells also have key regulatory roles in spermatogenesis through Sertoli cells [23,24]. Therefore, investigating waveform protein and androgen changes after busulfan treatment is valuable for elucidating the toxicity mechanisms of busulfan.

Therefore, this study investigated the effects of busulfan in a mouse model to determine its disruption mechanisms and identify ways to minimize the toxic effects.

## Materials and methods

The Methods section explains the experimental animals, methods, and data analysis used in this work.

### Animals

This study used six to eight weeks old, healthy, reproductively normal, male IRC mice purchased from Beijing Viton Lihua Laboratory Animal Technology Company. (China). The mice were housed at 20–25 °C under natural light conditions and allowed to eat and drink freely; the bedding was changed once per week.The mice were divided into one control (n = 9; 0 hours) and eight treatment (12, 24, and 36 hours and 3, 5, 7, 14, and 21 days; n = 9 per group) groups.

### Ethical statement

The Ethical Review Committee of Experimental Animals at the Institute of Animal Husbandry and Veterinary Medicine, Chinese Academy of Agricultural Sciences, Beijing, China, approved this study’s experimental methods (No. IAS2019−13). All efforts were made to reduce the number of animals used in this study and to ensure optimal conditions of well-being before, during and after each experiment. Mice were observed daily for general well-being and their weight was measured weekly.

### Busulfan treatment

The busulfan (Sigma-Aldrich, B1170000, USA) solution was prepared by dissolving 12 mg of busulfan powder in 1 mL of DMSO(Sigma-Aldrich, D2650,USA) and then adding 1 mL of physiological saline. The final busulfan concentration was 6 mg/mL. The dosage of busulfan is based on the previous research results of our team [12].

In total, 72 experimental mice and 9 control mice received testicular injections of busulfan solution (experimental groups) or DMSO solution (control group). The busulfan injection concentration was 6 mg/mL based on the study by Ruyi Li et al [14].

In the busulfan experimental groups, the mice were anesthetized by intraperitoneal injection of 2% sodium pentobarbital at a dose of 60 mg/kg body weight. Then, the scrotal area was disinfected with 75% medical alcohol, based on the blood vessel distribution on the surface of the testis, the blood vessels on the white membrane of the testis were avoided, and the injection entered the testis directly through the skin of the scrotum. Pressure was applied to the syringe to inject the busulfan solution (6 mg/mL) into the testis. In the control group, the mice were anesthetized and injected following the same procedure as the experimental group, but a DMSO solution of an equivalent volume was used instead. The same treatment was applied to the contralateral testis. The treated mice were kept in a single cage and observed for performance. After collecting blood from the posterior vein of the eye socket, euthanize mice with cervical dislocation, remove bilateral testicles and epididymis, and immediately fix them in tissue fixative.

The injection dose was determined based on the body weights of the mice, calculated as V = M, where V is the busulfan injection volume in μL, and M is the body weight of the mouse in grams.

### Testes and epididymis weights

After euthanasia and execution, the abdominal cavity of the mice was opened with scissors, and the testes and epididymis were quickly removed and weighed on an electronic balance.

### Sperm concentration measurements

The epididymis was placed in a 1.5 mL centrifuge tube containing 1 mL of saline, and the epididymal tail tissue was shredded with ophthalmic scissors to enable the release of epididymal sperm from the epididymal tail. The centrifuge tubes were kept at a constant temperature in a 37 °C water bath for 20 min to allow sufficient sperm to float out of the epididymal tail. Next, a coverslip was added to the counting cell of a clean and dry hemocytometer plate, and 10 μL of sperm suspension was pipetted onto the edge of the coverslip with a pipette so that the sperm suspension slowly infiltrated and filled the counting cell to prevent air bubbles. After standing for 5–10 min, the sperm were counted using a microscope (Leika, DM300, Germany).

### Sperm malformation rate

First, 500 μL of sperm suspension was placed in a new 1.5 mL centrifuge tube. Then, 50 μL of 2% eosin solution was added, mixed thoroughly, and stained for 1 min. Next, 10 μL of the mixture was pipetted onto the adherent slide with a pipette and applied evenly with the tip of the gun. The sample was air-dried under natural conditions, fixed with a methanol solution for 5 min, then the slides were sealed with neutral gum and air-dried at room temperature. The prepared sections were examined under a microscope (1000 × , oil) for sperm morphology, and the structurally intact and malformed sperm (divided into head and tail malformations) were counted. The main categories of malformed sperm were: fat-headed, round-headed, double-headed, headless, bent tail, curled tail, broken tail, and double-tailed [25–27]. Five hundred spermatozoa were examined per mouse. 81 mice were statistically analyzed.

### Paraffin tissue section preparation

The fixed tissues were placed in a gradient alcohol solution as follows: 50% ethanol (1.5 h), 75% ethanol (1.5 h), 85% ethanol (1 h), 95% ethanol (1 h), anhydrous ethanol I (30 min), and anhydrous ethanol Ⅱ (30 min). Then, they were incubated as follows: xylene:anhydrous ethanol mixture (1:1; 1 h), xylene I (1 h), xylene II (1 h). After embedding, the samples were cut into 5-μm thick sections by a paraffin slicer, placed in warm water (~40 °C) for spreading, and then baked in a thermostatic oven at 55 °C for 5 h after patching with adhesive slides to ensure the tissue sections fit completely on the slides.

### Hematoxylin and eosin (H&E) staining

The testicular paraffin sections were dewaxed by incubations in xylene I (30 min) and xylene Ⅱ (30 min), followed by 5 min incubations in xylene: anhydrous ethanol (1:1), anhydrous ethanol I, anhydrous ethanol II, 95% ethanol, 85% ethanol, 75% ethanol, 50% ethanol, and then distilled water. Next, the samples were stained with hematoxylin for 3 min, washed twice with distilled water (3 min, then 5 min), and stained with hematoxylin for an additional 3 min. After, the samples were washed with distilled water (3 min), fractionated with differentiation solution (10 s), washed with distilled water (2 min), returned to blue with the blue return solution (20 s), washed with distilled water (2 min), stained with eosin (8 min), and then washed with distilled water (5 min). After the sections were stained with H&E, they were sequentially dehydrated with a gradient of alcohol as follows: 85% ethanol (1 min), 95% ethanol (1 min), anhydrous ethanol I (5 min), anhydrous ethanol II (5 min), xylene I (5 min), and xylene II (5 min). Finally, the slices were sealed with neutral resin and observed under an ortho-fluorescence microscope (Leika, DM300, Germany).

### Immunofluorescence staining

Paraffin sections were dewaxed as described in the methods section hematoxylin and eosin staining, then washed with distilled water for 5 min. Next, the samples were subjected to low-pressure repair using the citrate antigen repair method for 5 min, high-pressure repair for 15 min, then left at room temperature. After, the samples were washed 3 times (5 min per wash) in 10 × Tris-buffered saline (TBS) and then incubated with 3% bovine serum albumin (BSA; closed solution configuration) with 2% TritonX100 permeation solution for 20 min. Next, the samples were washed 3 times (5 min each) with 10 × TBS, followed by incubations with 3% BSA closed solution configuration (1–2 h) and the primary antibodies (4 °C overnight). The primary antibodies were: connexin 43 (Cx43) (America abcam; ab235282; 1:100), N-cadherin (Invitrogen, Life Technologies; 33–3900; 1:50), and vimentin (America abcam; ab8978 1:150). The next day, the samples were washed 5 times with 10 × TBST (5 min each) and incubated with secondary antibody for 2 h at room temperature, protected from light. The secondary antibodies were: Cx43 (IgG H&L, Abcam; 1:500), N-cadherin (IgG H&L, Abcam; 1:200), and vimentin (IgG H&L, Abcam; 1:200); Finally, the samples were washed 5 times with 10 × TBST (5 min each), stained with DAPI for 5 min in the dark, then blocked with a small amount of anti-fluorescent bursting agent. Images were acquired using ortho-fluorescence (Leika, DM300, Germany) and confocal microscopes (Leika, TCSSP8, Germany) .

### Immunohistochemical staining

The paraffin sections were dewaxed and repaired as described in methods sections hematoxylin and eosin staining and Immunofluorescence staining, respectively. Next, the samples were washed 3 times with 10 × TBS (5 min each), followed by incubation with a 3% BSA (closed solution configuration) with 2% TritonX100 permeation solution for 20 min. After, they were washed three times with 10 × TBS (5 min each) and incubated with 3% BSA (closed solution configuration) for 1–2 h. After, they were incubated with primary antibody (occludin, [America abcam; ab216327 1:200]) overnight at 4 °C. The next day, the samples were washed 5 times with 10 × TBST (5 min each), followed by incubation with the secondary antibody (IgG H&L, America abcam; 1:500) at room temperature for 2 h, then 5 washes with 10 × TBST (5 min each). DAB color was added and developed for 5 min, then tap water was added to terminate the color development process. Next, hematoxylin re-staining was performed for 20 s, followed by a tap water rinse (5 min). Then, the differentiation solution was added for 20 s, followed by a tap water rinse (30 s), anti-blue solution incubation (20 s), tap water rinse (5 min), then gradient dehydration. The films were sealed with neutral resin, and images were acquired using an ortho-fluorescence microscope (Leika, DM300, Germany).

### Enzyme-linked immunosorbent assay (ELISA)

Serum from three mice per group was collected for TNF-α, IL-6, and IL-1β detection (inflammatory factors). Their testes were also sampled to make testicular homogenates, from which the supernatant was used for androgen and androgen receptor detection assays.

Standard and sample wells were included on each plate; 50 μL of the standards were added to their respective wells from low to high concentrations (TNF-α: 0, 40, 80, 160, 320, 640 pg/mL; IL-1β and IL-6: 0, 7.5, 15, 30, 60, 120 pg/mL; androgen and androgen receptor: 0, 2, 4, 8, 16, 32 ng/mL). Blank control wells (diluent only) were also included. In addition, 5-fold diluted samples were added to their respective wells (10 μL of sample + 40 μL of diluent). The plate was gently shaken to mix, then 100 μL of the enzyme reagent was added to each well except the blank controls. The plate was sealed with sealing film and incubated at 37°Cfor 60 min. Then, the liquid was discarded, the wells were washed with 20 × diluted (with distilled water) wash buffer 5 times (30 s each), and the plate was pat-dried. Next, 50 μL of chromogenic agent A and 50 μL of chromogenic agent B were added to each well, the plate was gently shaken to mix, and the color was developed at 37°C for 15 minutes, after which 50 μL of the termination solution was added to each well. The absorbance (optical density) of each well was measured at 450 nm within 15 min of terminating the color reaction using a (Tecan, Infinite 200 Pro, Austria) .

### BTB ultrastructure

The testes of three mice per group were cut into 1-mm-long tissue blocks, fixed in 2% glutaraldehyde overnight at 4 °C, then rinsed three times with 0.1 M phosphate-buffered saline (PBS) for 15 min each. Then, the samples were fixed with 1.3% osmium acid for 2 h, followed by three rinses with 0.1 M PBS for 15 min each. After, the samples were dehydrated in gradient alcohol (30%, 50%, 70%, 90%, and 100% ethanol followed by acetone; 12 min per step), permeabilized and embedded with resin, and then polymerized at 60 °C for 48 h in a constant temperature drying chamber. Next, they were stained for 15 min with uranyl acetate, followed by 3 washes with water for 2 min each, incubation with 0.1 M NaOH for 1 s, and lead citrate staining for 10 min. Finally, the sections were examined and photographed using a transmission electron microscope (Hitachi,HT7700, Japan) .

### Statistical analyses

All data were statistically analyzed using GraphPad Prism 8, and each data is presented as mean ± SD of at least three independent experiments. The statistical significance between the two groups was estimated using aone-way ANOVA. All groups were compared with each other for every parameter. The p < 0.05 was considered statistically significant.

## Results

### Toxic effects of busulfan treatment on mice

#### Effects on the testes.

**Fig 1:**
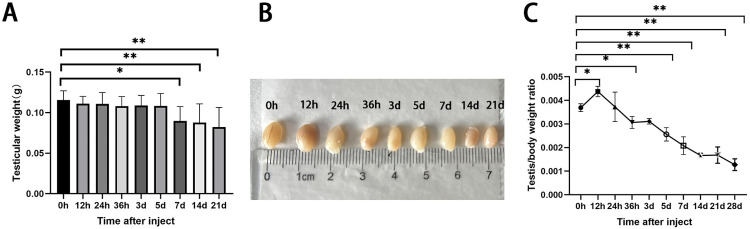
Effects of busulfan treatment (6 mg/kg) on the body and testes weights of mice over three weeks. (A) testicular volume, and (B) testicular weights decrease over time. (C) The testes-to-body ratio (testicular weight/body weight) significantly decreases with time.

In the busulfan-treated group (n = 6), the testicular volume gradually decreased in the treatment group (Fig 1A, F(8, 63)= 4.590, P < 0.01), and it’s weight decreased with time, with significant differences between hour 0 and days 7, 14, and 21 (Fig 1B). Finally, the testicular body ratio significantly decreased over time (Fig 1C, F(9, 30)= 44.96, P < 0.01), indicating a greater effect on the testes.

Groups: n = 6 per treatment; values are presented as means ± standard deviations; P-values are based on comparisons with the 0-hour measurement (control); * *p *< 0.05; ** *p* < 0.01.

#### Effects on the epididymis.

**Fig 2:**
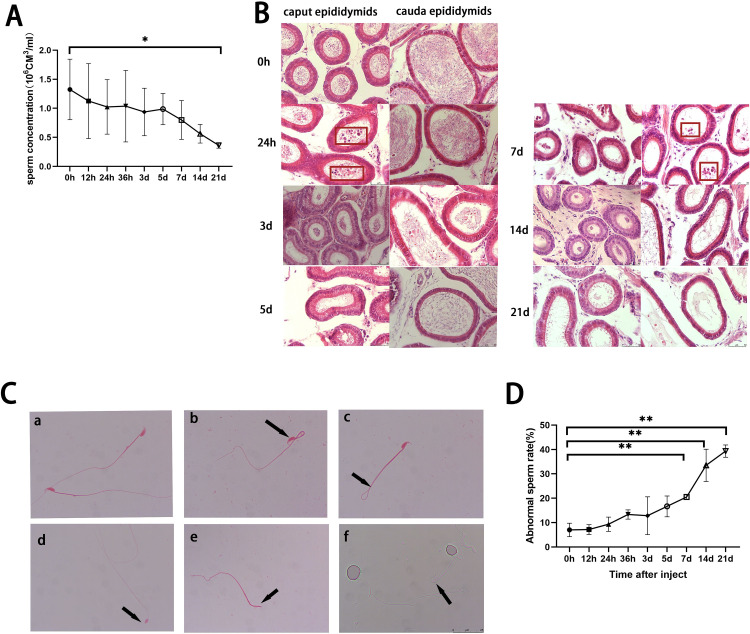
Effects of busulfan treatment (6 mg/kg) on the epididymis and sperm of mice over a three-week period. (A) The epididymal sperm concentrations decrease over time and significantly differ between hour 0 (control) and day 21 (*n *= 3 per group). (B) H&E staining of the epididymis (caput and cauda) over time (*n* = 3). Shed germ cells are identified by red rectangles after 24 hours and 7 days in the caput and cauda epididymis, respectively. Scale bar = 50 μm. (C) Representative images of sperm malformations (eosin stain; 1000 × magnification; *n* = 3): a) normal sperm; b) head-bent sperm; c) tail-bent sperm; d) hookless sperm; e) headless sperm; f) head-bent sperm; scale bar = 25 μm. (D) The sperm malformation rate increases over time (*n* = 3 per group).

In the busulfan-treated mice (n = 6), the sperm concentration of the epididymal semen (n = 3) also gradually decreased; hour 0 significantly differed from day 21 (Fig 2A, F (8,27) = 1.860, p = 0.1086). Furthermore, the head and tail of the epididymis of the control (n = 3) were filled with spermatozoa, but shed spermatocytes were visible in the head of the epididymis 24 hours after treatment (H&E; Fig 2B). After 3 days, the head and tail of the epididymis had significantly fewer spermatozoa and shed spermatocytes were visible in the tail. After 5 days, spermatozoa continued to decrease in the head and tail of the epididymis, and by day 7, the number of shed spermatocytes increased in the tail. Over time, increasing numbers of malformed spermatozoa were observed, including headless, head without a hook, bent-head, and folded-tail spermatozoa (n = 3; Fig 2C, D; F (8,18) =23.45, P < 0.01).

Values are presented as means ± standard deviations; * *p* < 0.05; ** *p* < 0.01

### Busulfan causes orchitis

**Fig 3:**
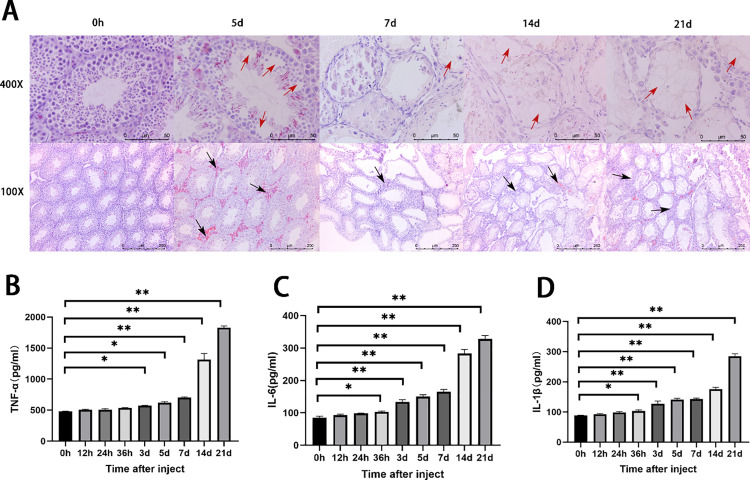
Busulfan treatment induces orchitis. (A) Hematoxylin and eosin staining of mouse testis tissue show gradual shedding of spermatogonia and decreases in the seminiferous tubule over time after busulfan treatment. First row: 400 × ; Second row: 100 × ; Scale bar = 250 μm. Red and black arrows represent vacuolation and inflammatory infiltration, respectively. ELASA results of the inflammatory factors, (B) tumor necrosis factor-alpha (TNF-α). (C) interleukin (IL)-6, and (D) IL-1β; the concentrations for all factors significantly increase over time.

Fig 3A presents the H&E staining results of the testicular tissue sections (n = 3). At 0 hours (control group), the seminiferous tubules were regularly arranged (100×), and the germ cells were closely and regularly arranged (400×). However, busulfan treatment induced gradual shedding of spermatogonia and a decrease in the seminiferous tubule. After 5 days, inflammatory cell infiltration between the seminiferous tubule (100×) could be seen, some spermatogonia were shed, and vacuoles appeared in the lumen of the ducts (400×). After 21 days, almost no spermatogonia were observed in the lumen.

Fig 3BD presents the ELISA results (n = 3); the serum TNF-α (F (8,18) =535.6, P < 0.01), IL-1β(F (8,18) =481.0, P < 0.01), and IL-6 (F (8,18) =385.1, P < 0.01)concentrations gradually increased in the busulfan-treated group. The IL-1β and IL-6 concentrations were significantly higher 36 hours after treatment compared to the control, and the TNF-α concentration was significantly higher after 3 days, peaking at day 21, compared to the control. Together, these results indicate that busulfan treatment induced orchitis.

Values are presented as means ± standard deviations; *n* = 3 per group. * *p* < 0.05; ** *p* < 0.01.

### Busulfan treatment damaged the BTB

#### Effects of busulfan treatment on BTB-associated proteins.

**Fig 4:**
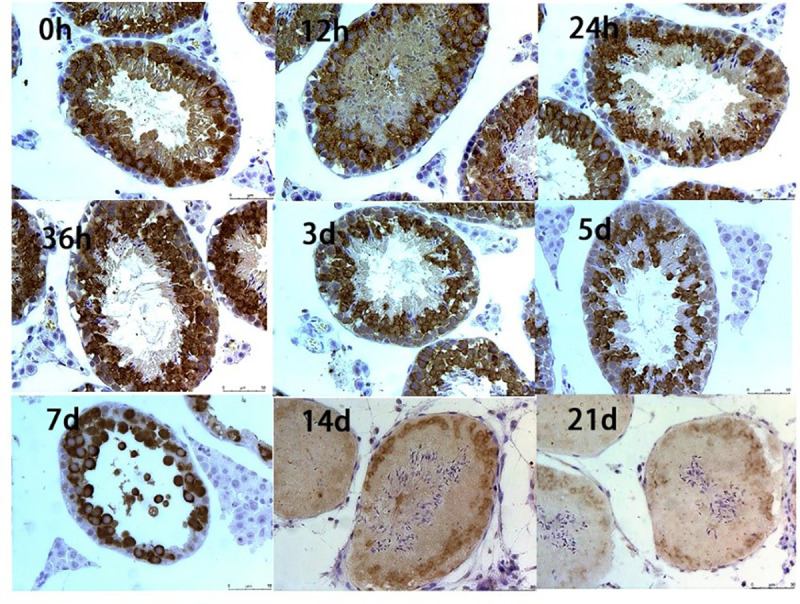
Effect of busulfan treatment on the tight junction protein, occludin. Occludin-positive signals around the spermatocytes decrease over three weeks. The brown signal represents the positive signal of occludin, and blue signal represents hematoxylin staining of the nuclei. (*n* = 6 per group; Immunohistochemistry staining; 400 × ; Scale bar = 50 μm).

Immunohistochemical detection of the tight junction-related protein, occludin, in testicular sections showed occludin-positive signals around the spermatocytes (n = 6; Fig 4). After 3 days, vacuoles appeared in the seminiferous tubule, and occludin-positive signals weakened; the signal range tended to contract. After 5 days, the number of vacuoles in the tubules increased, and the signal range contracted further. After 7 days, the positive signal range remained limited, but the tubular lumen became larger. After 21 days, the positive signals in the tubular lumen essentially disappeared, and fewer faint discontinuous signals were observed near the basement membrane.

**Fig 5:**
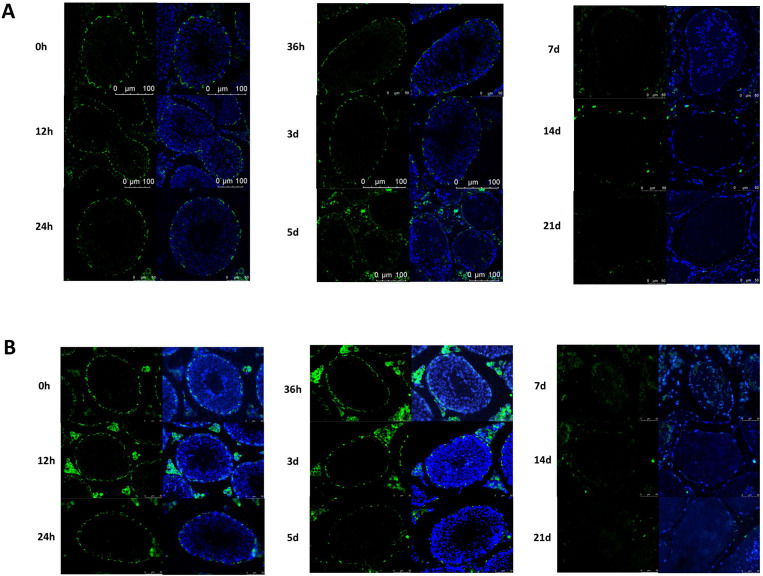
Effects of busulfan treatment on CX43 and N-Cadherin. The gap junction protein CX43 (A) and the ectoplasmic specialization-related protein N-Cadherin (B) were detected by immunofluorescence assay. Both positive signals decrease over three weeks. Control: 0h. Green represent positive signals for CX43 and N-Cadherin, and blue represent positive signals for DAPI staining of the nuclei, respectively. (*n* = 6 per group; Immunofluorescence staining; 400 × ; Scale bar = 50 μm).

Fig 5A presents the immunofluorescence staining results of testicular tissue sections for the gap junction protein, CX43 (n = 6). Positive CX43 signals were observed near the base of the seminiferous tubules, which surrounded the germ cells and Sertoli cells, separating them from the basement membrane myxoid cells. After busulfan treatment, the CX43 signal gradually weakened, and the nuclei gradually decreased. The positive signal distribution was discontinuous after 36 hours. However, after 5 days, the positive signal in the seminiferous tubule was notably weak and completely discontinuous, the number of nuclei significantly decreased, and the lumen of the tubule became larger. After 7 days, only a few positive signals near the basal layer remained, and the signal intensity and the number of nuclei further decreased; empty tubes also appeared. After 21 d, the positive signals in the seminiferous tubule essentially disappeared.

Fig 5B presents the immunofluorescence staining results for the ectoplasmic specialization-related protein (n = 6), N-cadherin, in the testicular tissue sections. Initially, N-cadherin-positive signals were identified around spermatogonial stem cells and Sertoli cells at the basal part of the seminiferous tubule. Five days after busulfan treatment, the N-cadherin positive signals decreased, the number of nuclei decreased significantly, and the lumen of the tubules became larger. After 7 days, only a few positive signals remained near the basal layer, the number of nuclei decreased further, and large empty tubules appeared. After 21 days, the N-cadherin positive signals in the seminiferous tubule essentially disappeared.

### Busulfan treatment damages the BTB ultrastructures

Immunofluorescence and immunohistochemical assays of testicular tissue sections showed that busulfan treatment reduced BTB-related proteins and their distribution became progressively discontinuous. Additionally, the number of cells in the seminiferous tubule decreased progressively.

**Fig 6:**
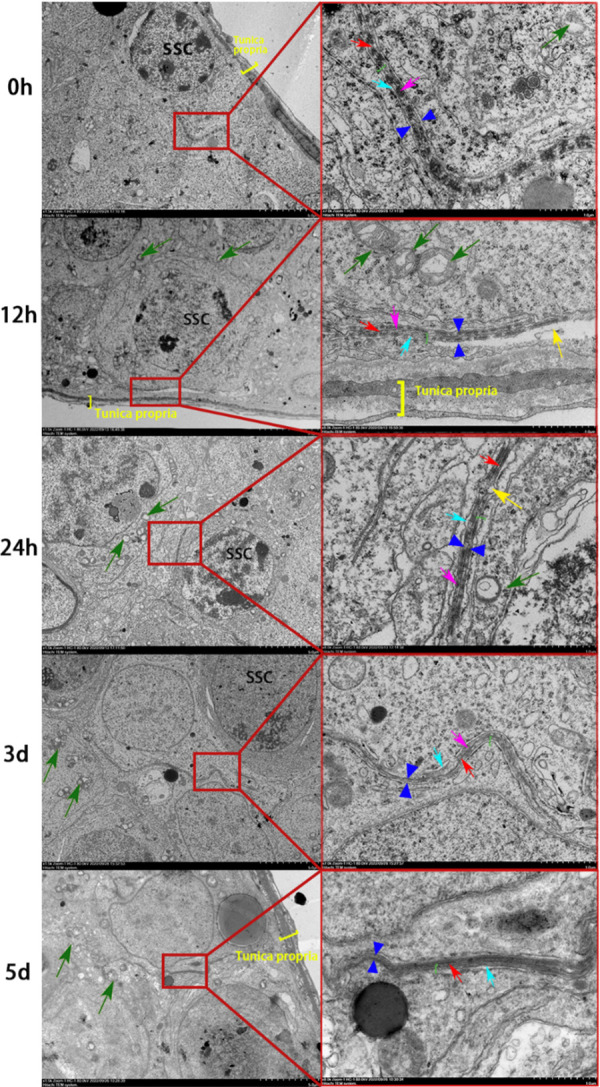
Effects of busulfan treatment on the ultrastructure of the blood-testis barrier (BTB). The fine structures of the BTB are progressively damaged over three weeks. Left side: 1500 × magnification (scale bar = 5 μm). Right side: a magnified view of the image on the left; 7000 × magnification (scale bar = 1 μm). Red arrows indicate tight junctions, green arrows indicate mitochondria, green brackets indicate basal ectoplasmic specialization, yellow arrows indicate vacuoles, and yellow brackets indicate the basement membrane.

We also analyzed the ultrastructure of testicular tissue sections using transmission electron microscopy to evaluate the effects of busulfan on the fine structures of BTB (n = 3; Fig 6). Typical structural features of the BTB were observed in the control (0 hours). For example, tight junctions and basal ectoplasmic specialization coexisting near the basement membrane bound by the relative plasma membrane were observed, as were regularly arranged bundles of actin filaments visible between the relative plasma membrane and the endoplasmic reticulum, with a small number of oval mitochondria around the adjacent cytoplasm and distinct mitochondrial cristae. Twelve hours after treatment, vacuoles appeared in the cytoplasm adjacent to the BTB, and the number of surrounding mitochondria increased, but the morphology was essentially normal. After 24 hours, vacuoles appeared next to the BTB, the surrounding mitochondria swelled and aggregated, and the cristae were not apparent. After 3 days, the BTB narrowed, and the mitochondria were vacuolated. Finally, after 5 days, the structure of the BTB was blurred, and the actin filament bundles were indistinguishable.

SSC: Spermatogonial stem cell. *n* = 3 per group.

### Effects of busulfan treatment on Sertoli cells

#### Busulfan treatment damages the cytoskeleton.

**Fig 7:**
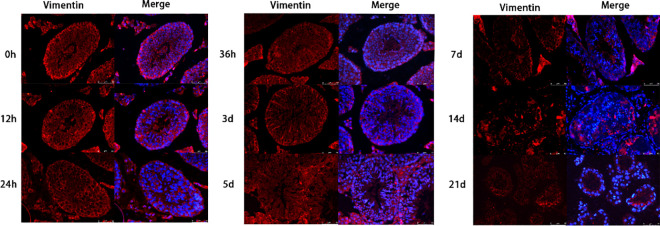
Effect of busulfan treatment on vimentin, a supporting cytoskeletal protein. Positive vimentin signals decrease over three weeks. Red and blue represent vimentin and DAPI staining of the nuclei, respectively. (*n* = 6 per group; Immunofluorescence staining; 400 × ; Scale bar = 50 μm).

Busulfan treatment caused orchitis, disrupted the integrity of the BTB, threatened the stability of the spermatogenic niche environment, and caused enlargement of the lumen of the seminiferous tubule. The enlarged lumen was caused by the shedding of spermatogenic cells and the shrinkage of Sertoli cells. Therefore, we detected vimentin, a supporting cytoskeletal protein, by immunofluorescence in the testicular tissues to further investigate the damaging effects of busulfan on Sertoli cells (n = 6; Fig 7).

In normal conditions, vimentin was diffusely distributed in the lumen of the seminiferous tubule with a strong fluorescent signal. Also, at all-time points, the spermatogonia signal was regularly scattered in the background and surrounded by the vimentin signal. Three days after busulfan treatment, the positive signals for vimentin weakened, the number of cells decreased, and the lumen of the ducts became larger. After 5 days, the positive signal further weakened and became unevenly distributed. After 7 days, only a few faint positive signals were observed, and the seminiferous tubule was wrinkled.

#### Effects of busulfan treatment on androgens and androgen receptors.

**Fig 8:**
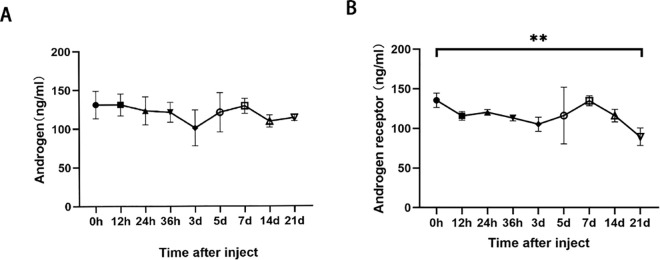
Effect of busulfan treatment on androgens and androgen receptors. Enzyme-linked immunosorbent assay results for A) androgens and B) androgen receptors. The androgen concentration remains stable after busulfan treatment, but the androgen receptor concentration significantly decreases 21 days after treatment. Values are presented as means ± standard deviations; n = 3 per group. * *p* < 0.05, ** *p* < 0.01.

Androgens are important regulators of spermatogenesis. We detected a decrease in the number and quality of spermatozoa after busulfan treatment. Thus, we analyzed the androgen levels in testicular homogenates (n = 3), but significant changes were not observed (Fig 8A, F (8,18) =1.220, P = 0.3422). We also analyzed the androgen receptor levels in the sertoli’s cells of the treated mice (n = 3), finding that the levels gradually decreased with the treatment time and were significantly lower after 21 days (Fig 8B, F (8,18) = 3.088, P = 0.0224). These results suggest decreased androgen receptor concentrations affected androgen function after busulfan treatment.

## Discussion

Understanding the physiological basis of spermatogenesis and maturation, enhancing the reproductive efficiency of breeding males, and achieving reproductive control are important research areas, especially for those preparing for SSC transplantation [28]. Busulfan is a common treatment option, but it has safety risks due to its highly toxic side effects. This study verified that busulfan damages the testes, induces orchitis from the inflammatory perspective, and damages Sertoli cells and BTB microstructures. These findings reveal some of the busulfan damage mechanisms, which can help improve the efficiency of recipient preparation for SSC transplantation.

Overall, testicular weight decreased after testicular injections with busulfan. However, the testicular weight increased significantly 12 hours after the injections, contrary to the overall trend and inconsistent with the fact that busulfan damages the testes. However, this result might be related to the immune response. Presumably, the testicular tissue underwent pharmacological edema after the busulfan injection; thus, capillary dilatation and body fluids entered the tissue interstitium, resulting in a temporary increase in testicular weight. The epididymis is an important organ for sperm maturation, and the effect of busulfan on the testes can be determined by testing the epididymis. The results suggest that the treatment damaged testicular tissue and affected spermatozoa development and maturation.

Moreover, the present study evaluated testicular sections after busulfan injection, finding inflammatory cell infiltration in the interstitium and a gradual decrease in the lumen of the seminiferous tubule ducts, vacuolation of the wall tissue, and shedding of spermatogenic cells. The study further showed the levels of inflammatory cytokines TNF – α, IL-1 β, and IL-6 gradually increased, and even exceeded the physiological upper limit after 3 days of treatment, resulting in an inflammatory phenotype. A study of autoimmune orchitis in mice also reported inflammatory cell infiltration, progressive reduction of the lumen of the seminiferous tubule, and shedding of spermatogonia in testicular tissue sections [13,29], supporting our conclusion that the busulfan treatment induced orchitis. There is research that proves the inflammatory factor IL-6 and its receptor IL-6R, promote apoptosis of spermatogenic cells and induce autoimmune orchitis [30]. Similarly, our study demonstrated that busulfan treatment induced orchitis and damaged spermatogenic cells and spermatogenic functions through a prolonged inflammatory response from both molecular and cellular perspectives. Previously, busulfan toxicity was mainly attributed to oxidative stress; thus, few studies on inflammatory mechanisms have been conducted. Our results indicate that busulfan damaged spermatogenesis by inducing inflammation, providing a different perspective studying busulfan damage to physiological functions. Inspired by the phenomenon of inflammatory infiltration, we thought that busulfan’s testicular injury is caused by inflammation. We conducted inflammatory factor testing and demonstrated for the first time that busulfan induces testicular inflammation damage to spermatogenesis function. This provides important theoretical support for the scientific use of busulfan in clinical practice and reduces its side effects.

A stable microenvironment is a prerequisite for efficient spermatogenesis, and the BTB structure, comprising tight junctions, gap junctions, ectoplasmic specialization, and bridging granule junctions, plays a key role in maintaining the stability of the spermatogenic environment [31]. After testicular busulfan injections, we found the occludin (a tight junction-related protein), CX43 (a gap junction-related protein), and N-Cadherin (a basal ectoplasmic specialization-related protein) content in the BTB structure gradually decreased, as did the structure’s continuity, these proteins are commonly used as marker proteins for BTB, these results all demonstrate that busulfan further damages the BTB structure by reducing the content of BTB protein. Environmental toxicants, such as bisphenol A (BPA) and cadmium, damage the BTB, downregulating relevant protein levels, thus, impairing spermatogenic function [32], which corroborates our conclusion that busulfan treatment induced orchitis, damaged BTB-related proteins, and destroyed the BTB. Additionally, previous studies reported that sodium fluoride and sulfur dioxide caused the loss of actin bundles and damaged the BTB ultrastructure [33], which greatly inspired this study. Therefore, to clarify the mechanisms of spermatogenic function damage by Busulfan we used transmission electron microscopy to evaluate the BTB structure, finding that the actin filament bundles were regular and tightly structured in the control samples (0 hours) and a small number of large, oval-shaped mitochondria with well-defined structures, such as matrix and cristae, were observed around them. It is hypothesized that BTB structural damage disrupts the stable environment and impairs the connection between germ cells and Sertoli cells, affecting spermatogenesis. In this study, we observed a disrupted BTB structure via transmission electron microscopy after busulfan treatment at the ultrastructural level. Although we also used immunofluorescence techniques to detect BTB damage, these assays were performed at a later timepoint than electron microscopy (5 days vs.12 hours); thus, the determination was primarily inferred.

Mitochondria provide energy to cells through the production of adenosine triphosphate by oxidative phosphorylation and energy for inflammatory signaling and transmission [34]. Environmental toxins, such as BPA and cadmium, can increase the production of reactive oxygen species in Sertoli cells, damaging mitochondria [35] and impeding mitochondrial fusion, division, and autophagy, which affects the health of an organism [36]. Using transmission electron microscopy, we observed smaller mitochondria and found that the mitochondrial cristae gradually disappeared and aggregated after busulfan treatment. These changes to the ultrastructure imply that busulfan damaged the mitochondria, disrupted the balance of mitochondrial fusion and division, affected the energy supply for spermatogenesis, and induced testicular inflammation.

Sertoli’s cells are the “nurse cells” that provide structural and functional support for spermatogenesis [37]. Together with microtubules, microfilaments and vimentin constitute the cytoskeleton of Sertoli cells, which are important for maintaining cell shape, differentiation, and motility, as well as ensuring intercellular signaling and material transport through cell junctions, and regulating normal germ cell differentiation [38]. In this study, we found that the fluorescence signal of waveform proteins was widely distributed in the seminiferous tubule canal walls, and the germ cells were spaced and arranged orderly. As a result, the germ cells lost the physical support and signal regulation of the Sertoli cells and cut off the material transport pathway; thus, they gradually separated from the Sertoli cells and shed. Studies have shown that waveform proteins provide physical support for BTB. Thus, damage severely affects the structure and function of the BTB, jeopardizing spermatogenesis and reducing sperm quantity and quality [39]. Previous studies have shown that busulfan can induce apoptosis in supporting cells [40]. Also, intact vimentin filaments regulate actin assembly [41]. Together, these previous studies support our findings; we observed a gradual weakening of the vimentin signal after busulfan treatment, suggesting damage to actin filament bundles in the BTB and the overall structure of the BTB. Therefore, we hypothesize that busulfan primarily acts on the Sertoli cells, damaging their actin filament bundles and vimentin and other skeletal components, causing atrophy; once physical support and regulation are lost, the spermatogenic cells gradually fall off, blocking spermatogenesis.

Androgens secreted by testicular interstitial cells play key regulatory roles in spermatogenesis, such as maintenance of the spermatogonial cell number, BTB integrity, meiosis, sperm cell adhesion, and sperm formation [42–44]. This study found that androgen levels remained stable after busulfan treatment, indicating that mesenchymal cells continued to steadily secrete androgen. Therefore, the main target of androgen receptors may not be in testicular mesenchymal cells. In contrast, the androgen receptor level decreased gradually with time, significantly differing from hour 0 on day 21. This result was similar to that of vimentin, suggesting a potential correlation between cytoskeleton and androgen receptor damage.

In conclusion, busulfan treatment induced testicular inflammation, damaging spermatogenic cells. Furthermore, BTB-related proteins (N-Cadherin, occludin, and CX43) and supporting cytoskeletal proteins (vimentin) deteriorated gradually after busulfan treatment, as did androgen receptors, suggesting structural damage to the BTB. Furthermore, transmission electron microscopy confirmed ultrastructural changes to the BTB over time, such as a gradually narrowing, a blurry structure, and vacuolation near the BTB, affecting the mutual support and regulation relationship between Sertoli and germ cells. Moreover, busulfan treatment caused a mitochondrial aggregation and division imbalance; thus, the mitochondria shrank, and cristae disappeared. Finally, busulfan treatment caused an inflammatory response and, thus, orchitis. Together, these results suggest that busulfan affects spermatogenesis by endangering the environmental stability and energy supply; therefore, its use should be carefully considered, especially for children and men wishing to bear children.
